# The Original Mouse Models of Glioblastoma: Analysis of Pathophysiological Characteristics of Transplanted Tumor Tissue

**DOI:** 10.17691/stm2025.17.5.03

**Published:** 2025-10-31

**Authors:** V.V. Kudelkina, M.V. Gulyaev, A.S. Khalansky, E.A. Miroshnichenko, O.V. Makarova, T.Kh. Fatkhutdinov, A.M. Kosyreva

**Affiliations:** Researcher, Laboratory of Neuromorphology; Avtsyn Research Institute of Human Morphology of Petrovsky National Research Centre of Surgery, 3 Tsurupy St., Moscow, 117418, Russia; Junior Researcher, B.V. Shvyrkov Laboratory of Psychophysiology; Institute of Psychology of the Russian Academy of Sciences, 13, Bldg. 1, Yaroslavskaya St., Moscow, 129366, Russia; PhD, Senior Researcher, Laboratory of Magnetic Tomography and Spectroscopy; Lomonosov Moscow State University, 1 Leninskiye Gory, Moscow, 119991, Russia; PhD, Collection Specialist; Avtsyn Research Institute of Human Morphology of Petrovsky National Research Centre of Surgery, 3 Tsurupy St., Moscow, 117418, Russia; Junior Researcher, Laboratory of Neuromorphology; Avtsyn Research Institute of Human Morphology of Petrovsky National Research Centre of Surgery, 3 Tsurupy St., Moscow, 117418, Russia; Junior Researcher, Department of Histology, Cytology, and Embryology; Peoples’ Friendship University of Russia named after Patrice Lumumba, 6 Miklukho-Maklaya St., Moscow, 117198, Russia; MD, DSc, Professor, Head of the Laboratory of Immunomorphology of Inflammation; Avtsyn Research Institute of Human Morphology of Petrovsky National Research Centre of Surgery, 3 Tsurupy St., Moscow, 117418, Russia; MD, DSc, Associate Professor, Chief Researcher, Laboratory of Growth and Development; Avtsyn Research Institute of Human Morphology of Petrovsky National Research Centre of Surgery, 3 Tsurupy St., Moscow, 117418, Russia; Head of the Department of Histology, Cytology, and Embryology; Peoples’ Friendship University of Russia named after Patrice Lumumba, 6 Miklukho-Maklaya St., Moscow, 117198, Russia; PhD, Head of the Laboratory of Neuromorphology; Avtsyn Research Institute of Human Morphology of Petrovsky National Research Centre of Surgery, 3 Tsurupy St., Moscow, 117418, Russia; Professor, Department of Histology, Cytology and Embryology; Peoples’ Friendship University of Russia named after Patrice Lumumba, 6 Miklukho-Maklaya St., Moscow, 117198, Russia

**Keywords:** glioblastoma mouse model, morphology, gene expression, intratumor immune response

## Abstract

**The aim of this study** was to morphologically, molecularly, and immunologically characterize two new transplantable glioblastoma (GB) tissue models, designated M2 GB and M6 GB.

**Materials and Methods:**

Two new chemically induced, easily transplantable tissue mouse models of high-grade glioma have been created and characterized. M2 GB and M6 GB tissues were orthotopically transplanted to immunocompetent C57BL/6 mice. The clinical and morphological characteristics of tumor growth, as well as the intratumoral immune response and target gene expression were assessed.

**Results:**

Clinical manifestations of M2 GB and M6 GB growth in mice include motility disorders, cachexia, and priapism. Morphologically, M2 GB and M6 GB are characterized by diffuse proliferation, cellular and nuclear polymorphism, and high mitotic activity with pathological mitotic patterns corresponding to the aggressive nature of the mentioned tumors. Both tumors were significantly infiltrated with CD3^+^ T lymphocytes (~32%) and F4/80^+^ macrophages (~28–50%). M2 GB showed a higher content of F4/80^+^ macrophages compared to M6 GB. The *Cdkn2a*, *S100b*, *Mki67*, *Pten*, *Vegfa*, *Hif1a*, *Sox2*, *Abcb1*, and *Gfap* genes were overexpressed in both tumors. Expression of the *Cd133*, *Tp53*, and *Pdgfra* genes was increased in M2 GB. High expression of *Pi3k* and *Gdnf* was seen in M6 GB. Expression of *Cd44*, *Pi3k*, *Hif1a*, *Gdnf*, and *Egfr* was higher in M6 GB tissues compared to M2 GB, whereas expression of *Cdkn2a*, *Tp53*, *Cd133*, and *Pdgfra* was higher in M2 GB tissues compared to M6 GB.

**Conclusion:**

The M2 GB and M6 GB models of transplanted tissues reproduce key characteristics of human GB, including similar intracellular immune profiles, clinical and morphological features, and gene expression patterns, which are important for further research in neurological oncology. These models can be used to develop diagnostic and treatment methods and to study tumor genesis.

## Introduction

Glioblastoma (GB) is the most aggressive tumor in the central nervous system in adults. Despite improved short-term survival, long-term treatment effects are still poor, and tumor recurrence rate is almost 100% [1]. Understanding the mechanisms of tumor initiation, progression, and evolution is critical for therapeutic approaches development. For this purpose, relevant preclinical models of GB are required. Mouse model is the most commonly used model for preclinical studies [2]. Allogeneic, xenograft, and genetically modified mouse tumor models are known, each having its advantages and limitations. Genetically modified models are expensive and time-consuming to create, and gene expression may be suppressed in later generations of animals [3]. Xenografts do not reflect early stochastic carcinogenesis, and the molecular biological characteristics of the host organism and tumor differ. In this model, it is not possible to assess the impact of the immune system on carcinogenesis, therapy, and immunotherapy [4]. Allogeneic models do not represent the many human tumor types with characteristic driver molecular abnormalities [5], but they are easy to model and use in large series of experiments. In translational studies of human brain glioma, a number of key factors should be considered: the molecular, biological, and pathophysiological characteristics of the glioma model and the model organism, as well as the local and systemic immune response and the clinical pattern of tumor growth. The response of the GB model to treatment should be similar to the human tumor response, and the tumor should be chemically and radiologically resistant [6]. Available GB models have unique characteristics that should be taken into account when planning experiments, though currently there is no model that accurately reproduces human GB [2, 7–9]. Low reproducibility of successful results in preclinical studies, the difficulty of their correct interpretation and translation into clinical practice emphasize the importance of the correct selection of experimental models.

**The aim of this study** was to morphologically, molecularly, and immunologically characterize two new transplantable glioblastoma tissue models, designated M2 GB and M6 GB.

The study demonstrated that transplantable tumor tissue models have advantages compared to commonly used cell models: a heterogeneous cellular composition and gene expression more similar to human tumors [10, 11].

## Materials and Methods

The study was conducted on 54 mature male house mice (*Mus musculus*) of the C57BL/6 line (20–22 g). The animals were allocated as follows: 22 mice for primary M2 GB and M6 GB tumor tissue collection; 14 for tumor tissue revitalization; 12 for the core experiment, including 6 for M2 GB and 6 for M6 GB; and 6 intact animals. The animals were kept in cages with a 12-hour light/dark cycle with unlimited access to food and water. Surgical and culture procedures were performed under aseptic conditions. Animal experiments were carried out in accordance with the European Convention for the Protection of Vertebrate Animals used for Experimental and Other Scientific Purposes (Strasbourg, 1986). The study was approved by the Bioethics Commission (Protocol No. 29(5) dated November 8, 2021) of the Avtsyn Research Institute of Human Morphology of Petrovsky National Research Centre of Surgery (Russia). Animals with the first clinical signs of tumor growth (decreased activity, paralysis, weight loss, and weakness) were euthanized [12].

### Induction of primary tumors with a carcinogen

Primary tumor tissues were obtained after trocar- assisted implantation of 1 mg of the solid carcinogen 7,12-dimethylbenz[a]anthracene (DMBA) (Thermo Fisher Scientific, USA) into the right cerebral hemisphere of house mice (*Mus musculus*; n=22) under intraperitoneal anesthesia using Zoletil 100 of 0.25 mg/animal (Virbac, France) with Xylanite of 0.5 mg/animal (Interchemie Werken, Netherlands). 60–90 days after DMBA implantation, a range of central nervous system (CNS) tumors developed in mice. Tumor tissues with morphological features of GB (M2 GB and M6 GB) were selected for primary sequential transplantations (8– 10 transplantations).

After 8–10 consecutive transplantations of primary tumor tissue into mice, stable M2 GB and M6 GB tissue models with similar morphology and growth latency periods were obtained. The transplanted M2 GB and M6 GB tumor tissues strains were registered and are stored at –196°C in the cryostorage facility of the Avtsyn Research Institute of Human Morphology of Petrovsky National Research Centre of Surgery FSBSI. They are cataloged in the Registry of experimental tumors of the nervous system (http://ckp-rf.ru/usu/498710/). They are used in experimental neurological oncology.

### Revitalization and transplantation of M2 GB and M6 GB tissue samples after cryopreservation

Before the core study, the tumor tissues were revitalized. For this purpose, an ampoule containing tumor tissue was thawed in warm water (39°C) and centrifuged for 7 min at 250 g. Then, the supernatant was removed, and the pellet was resuspended in glutamine-free culture medium (PanEco, Russia). ~10 μl of GB tissue (~4·10^5^ cells) was injected intracerebrally using a syringe and needle (20G) with a stopper. The cells were transplanted into animals (n=14) under intraperitoneal anesthesia (Zoletil 100 of 0.25 mg/animal and Xylanit of 5 mg/animal).

### Transplantation of M2 GB and M6 GB tissue samples after revitalization to study the survival and progressing development of M2 GB and M6 GB tissue samples

Grown GB tissues at the final stages of growth were transplanted within an hour into animals of the main experimental group, as described below.

After mechanical dissociation (pipetting) of the tumor tissue, cell viability was quantified in ~10 μl tissue samples (n=12). Cell viability was assessed using a 0.4% aqueous trypan blue solution (Servicebio, China). Cells were counted immediately after staining using a Goryaev chamber (MiniMed, Russia) under a light microscope (Carl Zeiss, Germany). Cell viability was at least 98%.

Mechanically crushed tissues of M2 GB (n=6) and M6 GB (n=6) with a volume of ~10 μl (~4**·**10^5^ cells) were implanted into the brain of mice under intraperitoneal anesthesia with Zoletil 100 of 0.25 mg/animal and Xylanite of 0.5 mg/animal. The detailed procedure of tumor transplantation is described in the earlier publication [13]. However, in this case, the tumor was transplanted into the brain 2 mm to the right of the sagittal suture (sutura sagittalis) and 2 mm caudal to the coronal suture (sutura coronalis) to a depth of 2 mm in the corpus striatum region using a stereotaxic device (RWD, China); AP: +1 mm; ML: +2.0 mm lateral to bregma; DV: –2.0 mm relative to the skull surface. The injection rate was ~10 μl/10 s for both models.

***MR images of the mouse brain*** were obtained intravitally on day 16 for M2 GB and on day 23 for M6 GB using a 7T tomographic scanner (BioSpec 70/30 USR; Bruker BioSpin, Germany) with a gradient amplitude of 105 mT/m. Isoflurane (Laboratorios Karizoo, Spain) was used for anesthesia. Axial T1-weighted images were acquired 15 min after intraperitoneal injection of gadobutol contrast agent (Schering, Germany) of 15 mg gadolinium per animal. Slice thickness was 0.8 mm, and the number of slices was up to 32.

***Morphological examination of the brain of animals*** with GB was conducted at the terminal stages of tumor growth, specifically on days 17–19 for M2 GB and on days 24–26 for M6 GB after transplantation. This time frame corresponds to the onset of the first clinical symptoms in mice; death occurs within 1–2 days from the onset of the first clinical signs (decreased activity, paralysis, weight loss, and priapism). Animals were euthanized with Zoletil 100 of 10 mg/kg. Brains with GB (n=12) were fixed in 10% buffered formalin (BioVitrum, Russia). Tissue sections (7 μm thick) were prepared on a Microm HM 340 microtome (Thermo Fisher Scientific, Germany) and stained with hematoxylin and eosin (BioVitrum, Russia). Tissue samples for each GB strain were taken from at least 6 mice. Morphological changes were assessed using a light microscope (Carl Zeiss, Germany).

Gene expression was analyzed by real-time PCR. Total RNA was isolated using the RNeasy Plus Mini kit (Qiagen, USA) from 6 M2 GB tissue samples, 6 M6 GB tissue samples, and 6 intact brain samples (30 mg each). The samples were stored in RNA Later solution (Invitrogen, USA). The mRNA content was greater than 300 ng/μl (NanoPhotometer N50; Implen, Germany). cDNA was synthesized from the total RNA using the MMLV RT kit (Eurogen, Russia). PCR assay was performed using qPCRmix-HS SYBR reagents and the fluorescent intercalating stain SYBR Green I (Eurogen, Russia). Primers were selected using the Primer-BLAST online resource (USA) according to the generally accepted requirements. The selected primers (Table 1) were synthesized by Evrogen (Russia). The threshold cycle (Ct) method was used and relative gene expression was calculated according to the method [14] taking into account the recommendations [15]. The *Gapdh* gene was used as a control. mRNA expression was compared between M2 GB and M6 GB tumor samples and intact mouse brain tissue.

**T a b l e 1:** Sequence of primers used to determine the expression level of the corresponding gene

Gene	Forward primer	Reverse primer
*Cd44*	AGAAGGGACAACTGCTTCGG	TTGGAGCTGCAGTAGGCTG
*Cdkn2a*	TGGTCACTGTGAGGATTCAGC	TGCCCATCATCATCACCTGG
*Pi3k*	CCGCTCAGGGAGAGGAGTA	CCACTCTCAGCTTCACCTCC
*S100b*	GATGTCCGAGCTGGAGAAGG	CCTGCTCCTTGATTTCCTCCA
*Tp53*	TTCTCCGAAGACTGGATGACTG	CTGCTCCTTGATTTCCTCCA
*Mki67*	CCTGCCTGTTTGGAAGGAGT	AAGGAGCGGTCAATGATGGTT
*Pten*	GGACCAGAGACAAAAAGGGAGT	CCTTTAGCTGGCAGACCACA
*Vegfa*	TCCACCATGCCAAGTGGTC	AGATGTCCACCAGGGTCTCA
*Hif1a*	GATGTCCGAGCTGGAGAAGG	CTGTCTAGACCACCGGCATC
*Cd133*	GGAGCAGTACACCAACACCA	GTCTGTTTGATGGCTGTCGC
*Sox2*	AGGAAAGGGTTCTTGCTGGG	GGTCTTGCCAGTACTTGCTCT
*Pdgfra*	GTGCTAGCGCGGAACCT	CATAGCTCCTGAGACCCGC
*Gdnf*	GACCGGATCCGAGGTGC	GAGGGAGTGGTCTTCAGCG
*Mgmt*	GACCGGATCCGAGGTGC	GAGGGAGTGGTCTTCAGCG
*Abcb1*	CTCTTGAAGCCGTAAGAGGCT	AACTCCATCACCACCTCACG
*Gfap*	GGCTGCGTATAGACAGGAGG	CCAGGCTGGTTTCTCGGAT

The relative number of lymphocytes and macrophages in the tumor tissue (10^6^ cells; at least 5 samples for each GB strain) was assessed using a Cytomics FC 500 flow cytometer (Beckman Coulter, USA). Erythrocytes were lysed using OptiLyse C solution (eBioscience, USA). GB samples were incubated with antibodies to CD3- FITC (eBioscience, USA) and F4/80-PE (Miltenyi Biotec, Germany) for at least 30 min at room temperature. Samples without antibodies were used as controls for autofluorescence.

***Statistical data analysis*** was conducted using the Statistica 10.0 package (StatSoft Inc, USA). Experimental data were characterized using the median (Me) and interquartile range [Q1; Q3]. Statistical differences were determined using the Kruskall– Wallis multiple comparison test. Dunn’s test was used for pairwise comparisons. Values were considered statistically significant at p≤0.05.

## Results

The incidence of M2 GB and M6 GB formation was 95–100%. The average latent period of tumor growth in mice was 17–35 days for M2 GB and 23–34 days for M6 GB.

**Figure 1.**
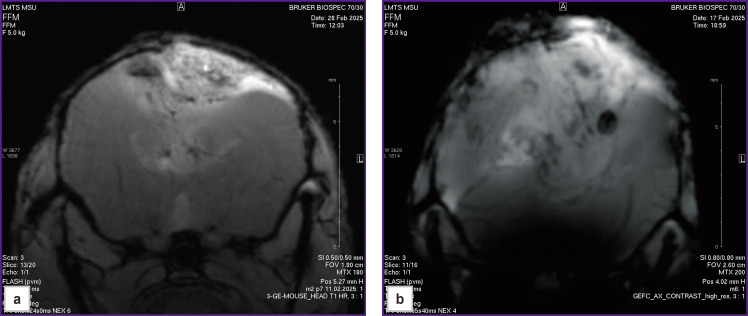
Axial T1-weighted magnetic resonance images of mouse brain sections with M2 (a) and M6 (b) glioblastoma

According to MRI of the brains of mice with M2 GB and M6 GB, the tumors were located in the right hemisphere; in the late stages, growth into the left hemisphere and a mass effect were observed: hemispheric asymmetry, compression with tissue deformation, and lateral displacement of midline brain structures (Figure 1).

**Figure 2.**
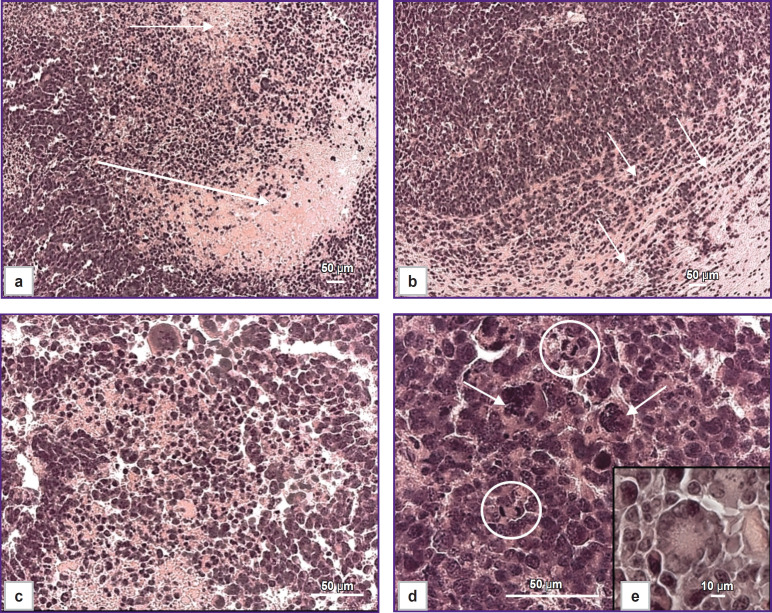
Histopathological features of the M2 glioblastoma model: (a) extensive necrosis and hemorrhage (*arrows*); (b) infiltrative growth at the tumor-brain interface (*arrows*); (c) tumor cells exhibiting signs of cell death and mitotic figures alongside hemorrhage; (d) hyperchromatic, polymorphic, and multinucleated giant cells (*arrows*) and mitotic figures (*circles*); (e) focal formation of rosette-like structures by tumor cells. Staining: hematoxylin and eosin

Morphologically, M2 GB cells (n=6) are large, exhibit high mitotic activity (Figure 2 (d)), and spread into the cortex and subcortical structures of the brain. The cells are polymorphic and atypical, with large nuclei, high cellularity, and a significant number of multinucleated giant cells (Figure 2 (c), (d)). Approximately 2% of mitoses were detected in the tumor, including pathological ones, which corresponded to two or more mitoses per 100 tumor cells. Approximately 2% of dying cells contained fragmented nuclei in the form of apoptotic bodies. Infiltrative growth (Figure 2 (b)), astroglial accumulation, and peritumoral edema were observed at the tumor–brain interface. Necrosis and hemorrhage were detected within the tumor parenchyma (Figure 2 (a)). Rosette-like tumor cell clusters (Figure 2 (e)) are a diagnostic feature of certain CNS tumors, such as medulloblastoma and neuroblastoma.

**Figure 3.**
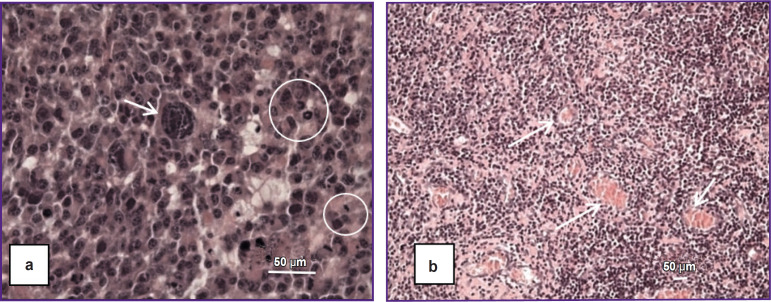

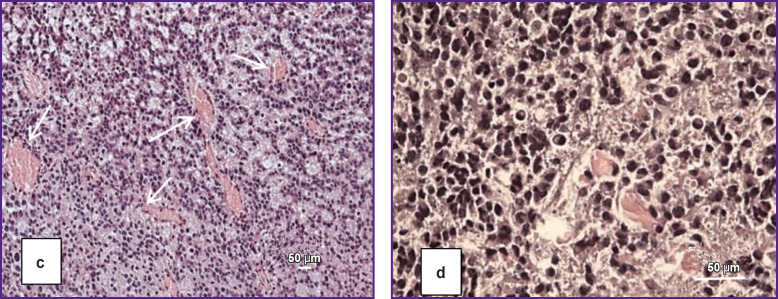
Histopathological features of the M6 glioblastoma model: (a) cellular and nuclear polymorphism, a giant multinucleated cell (*arrow*), and numerous mitotic figures (*circles*); (a)–(d) overall tumor hypercellularity; (b), (c) numerous blood vessels within the tumor parenchyma (*arrows*); (d) hyperchromatic, polymorphic tumor cells and nuclei. Staining: hematoxylin and eosin

The M6 GB tumor cells (n=6) are polymorphic, with narrow cytoplasmic margins, predominantly giant morphology, and multiple nuclei (Figure 3 (a), (d)). Mitotic figures, including pathological forms, accounted for approximately 3% (see Figure 3 (a)), equivalent to three or more mitoses per 100 tumor cells. Approximately 3% of cells were identified as apoptotic bodies. The tumor parenchyma contained numerous vessels with distorted lumens, microvascular proliferation, and hemorrhages (Figure 3 (b)–(d)), along with several necrotic foci. Tumor growth was characterized by infiltrative and perivascular patterns.

Both M2 GB and M6 GB tissues were infiltrated by CD3^+^ T lymphocytes and F4/80^+^ macrophages. The relative numbers of T lymphocytes and macrophages in the M6 GB group (n=5) were 32.01 [8.90; 33.60]% and 28.4 [14.8; 28.4]%, respectively. In the M2 GB samples (n=5), the macrophage count was statistically significantly higher (p=0.04) at 50.3 [49.4; 51.2]% compared to 28.4 [14.8; 28.4]% in the M6 GB group.

A comparative assessment of gene expression in the M2 GB and M6 GB models and intact brain tissue, alongside data from human GB [16–32], is presented in Table 2.

**T a b l e 2:** mRNA expression levels in M2 GB and M6 GB tumors compared to intact brain tissue

Gene	Function	Brain tissue (n=6)	GB М2 (n=6)	GB М6 (n=6)	^p^	Reference (human GB)
*Cd44*	Cell adhesion, neurotrophic factor	9 [4; 32]	7 [6; 11]	39 [36; 55]	0.002***	[16] ↑
*S100b*		126 [49; 897]	9155 [6851; 1429] ↑	1742 [1204; 3406] ↑	0.000*; 0.041**	[17] ↑
*Pi3k*	Angiogenesis, differentiation, transcription	45 [35; 70]	105 [87; 155]	301 [229; 900] ↑	0.000**; 0.007***	[18] ↑
*Vegfa*		51 [36; 423]	2109 [1242; 4244] ↑	8269 [8; 11 877] ↑	0.033*; 0.015**	[19] ↑
*Cdkn2a*	Cell cycle, apoptosis, transcription, lipid metabolism, neurogenesis	19 [3; 38]	68721 [43 328; 84 452] ↑	10 496 [9155; 15 291] ↑	0.000*; 0.047**; 0.010***	[20] ↑
*Tp53*		1141 [770; 1458]	93 397 [5336; 22 744] ↑	40 [25; 201] ↓	0.004*; 0.005**; 0.000***	[21]
*Mki67*		9 [2; 16]	4410 [1972; 7018] ↑	747 [539; 1285] ↑	0.000*; 0.032**	[22]
*Pten*		424 [272; 1011]	5301 [2633; 6966] ↑	6736 [4548; 16 931] ↑	0.000*; 0.000**	[23] ↑
*Hif1a*	Transcription	245 [133; 375]	2269 [1402; 2725] ↑	5876 [4951; 7018] ↑	0.006*; 0.000**; 0.046***	[24] ↑
*Sox2*		1029 [358; 2219]	5876 [3967; 7018] ↑	12 060 [10 322; 17 055] ↑	0.009*; 0.000**	[25] ↑
*Cd133*	Differentiation, proliferation, apoptosis	82 [35; 88]	168 [137; 218] ↑	36 [25; 42]	0.020*; 0.000***	[26]
*Pdgfra*	Growth factors, chemotaxis	71 [30; 296]	505 [276; 773] ↑	43 [29; 68]	0.009*; 0.000***	[27] ↑
*Gdnf*	Growth factors	3 [2; 5]	16 [9; 26]	1065 [586; 1376] ↑	0.000**; 0.001***	[28] ↑
*Mgmt*	DNA damage/repair	23 [2; 96]	30 [9; 73]	31 [16; 47]	>0.050	[29] ↑
*Abcb1*	Cellular transport	33 [27; 53]	302 [245; 426] ↑	596 [523; 968] ↑	0.002*; 0.000**	[30]
*Gfap*	Glioma-associated	281 [35; 1554]	2702 [1051; 5301] ↑	6736 [4548; 12 104] ↑	0.050*; 0.000**	[31] ↑
*Egfr*	Proliferation	14 [5; 26]	1.0 [0.4; 1.0]	27 [5; 110]	0.011***	[32] ↑

N o t e. Data are presented as Me [Q1; Q3]; Kruskal–Wallis and Dunn test; * brain and M2 GB; ** brain and M6 GB; *** M2 GB and M6 GB. GB — glioblastoma. ↑ and ↓— up and down regulation of gene expression, respectively.

## Discussion

Some studies noted that patients [33–36] and experimental animals [37–39] with brain tumors had a clinical pattern associated with decreased activity, paralysis, weight loss, and priapism. Similar symptoms in mice with M2 GB and M6 GB were observed in our study, indicating a terminal state in animals. The clinical manifestations of GB progression were associated with systemic metabolic alterations, including cachexia, coagulopathy, and signs of ischemic brain injury. Associated neurological symptoms included priapism and motor deficits.

Neuropathologists assign a tumor a high-grade malignancy based on 2 or more mitoses in the whole sample or 1 mitosis in a small biopsy [40]. This is consistent with the data on the high-grade malignancy of M2 GB and M6 GB received in this study.

Glioblastoma is an immunosuppressive tumor. Local and systemic immune dysfunction limit the efficacy of immunotherapy. Myeloid-derived suppressor cells and tumor-associated macrophages can inhibit T-cell infiltration and activation in gliomas [41]. Glioma cells and infiltrating immune cells evade immune surveillance by secreting immunosuppressive factors, such as IL-6, IL-10, TGF-β, and prostaglandin E2, whose expression is regulated by tumor-derived growth factors [42]. The difference in the abundance of T lymphocytes and macrophages between the M2 GB and M6 GB models is likely determined by the higher secretion of antiinflammatory cytokines by M2 GB cells.

Tumor-infiltrating stromal cells, including macrophages/microglia, often exert a more potent immunosuppressive effect than the tumor cells themselves. Immunosuppression impacts the clonal composition of tumor cells and modulates gene expression [43]. An increased content of T lymphocytes in a tumor indicates an inflammatory tumor status, and a high number of F4/80^+^ indicates an immunosuppressive background in the tumor [44]. HIV-mediated immunosuppression is associated with an increased incidence of gliomas and the progression of low-grade glioma to GBs, which evidences a significant contribution of the immunosuppressive microenvironment to glioma progression. In patients with GB and mice with GB, the number of CD4 T lymphocytes in the blood (systemic lymphopenia) in some cases may correspond to the number of CD4 T lymphocytes in AIDS. In lymphoid organs, a deficiency of T-cells is seen, whereas in the bone marrow, many naive T-cells are found. This phenomenon is characteristic not only of brain tumors but also of other types of tumors, but this is relevant only when they grow intracerebral [45, 46]. Compared with tissue models of M2 GB and M6 GB, macrophage populations in a cell tumor model, GL261 mouse glioma, are low (on average 5.6%) [47]. According to other sources [48], this model is heavily infiltrated by immune cells. In humans, GB has variability in the content of immune cells. The expression of genes associated with the immune response, as well as the infiltration of macrophages, CD3^+^ and CD8^+^ T-cells increased during the initial phase of GB growth in mice and then decreased as tumor grew. This indicates a “correction” and “deprivation” of the immune response by the tumor [49].

The presence of CD3^+^ T-cells in GB samples indicates an immune response to the tumor. Treatment- induced release of tumor neoantigens are released, significantly increasing the CD3^+^ cell count. However, it has been suggested that during GB relapse, the CD3^+^ T-cell response may be limited due to a lack of antigens. According to some researchers, the presence of intratumoral CD3^+^ cells before treatment positively correlates with patient survival [50], but others have not confirmed this association [51]. Most human GB cases exhibit low CD3^+^ infiltration, consistent with the characteristic “cold” immune profile of the tumor. Approximately 25% of human GBs have moderate or high CD3^+^ infiltration [52], similar to the M6 GB model. Therefore, the M6 GB model represents a valuable tool for advancing immunotherapy strategies for this specific group of GB patients.

Compared with cell lines and chemically induced primary tumors, transplanted tumor tissue is more carcinogenic and exhibits more aggressive growth and rapid macrophage polarization to the M2 phenotype. Most immunosuppressive cytokines, enzymes, checkpoint ligands, cell surface molecules, and signal pathways are overexpressed in glioma stromal cells and macrophages/microglia, but not in tumor cells [49]. M6 GB and M2 GB are heavily infiltrated by tumor- associated lymphocytes and macrophages. This finding requires further study. Gene expression should aim to separately analyze gene expression in M2 GB and M6 GB tumor cells, as well as in the surrounding immune cells.

Mutations in the *Hras*, *Pten*, *Pi3k*, *Mdm2*, *Tp63*, *Esr*, *Pgr*, and *Her2* genes, loss of *Cdkn2a*, *Igf*, and *Akt*, as well as MYC phosphorylation have been identified in DMBA-induced breast, blood, and skin tumor models [53–55]. Furthermore, these tumors are characterized by activation of *Egfr* and *Mki-67*, wild-type p53, low levels of mRNA and CDKN1A and PTEN proteins, and decreased expression and activity of the IDH1/2 enzyme, one of the main markers of gliomas [56].

Human GB is almost always classified as IDH- wildtype. It is characterized by activating alterations in the *EGFR* gene in approximately 57% of cases and in the *TERT* promoter in over 70–80% of cases. Notably, *EGFR* activation is less frequent in giant cell GB and gliosarcoma. Our study demonstrated that the M2 GB model, which contains a significant number of giant cells, exhibits consistent *Egfr* expression. In contrast to GB, IDH-mutant astrocytomas often harbor wild-type *EGFR* and *PTEN* genes, along with *MGMT* promoter methylation. These astrocytomas also frequently feature mutations in the *TP53* and *ATRX* genes and the *TERT* promoter [57, 58]. Discrepancies between the *MGMT* promoter methylation status and treatment response in some patients may be attributed to a lack of correlation between *MGMT* methylation and actual *MGMT* protein expression levels in GB [59].

Aggressive tumor cells, especially poorly differentiated cells [60, 61], upregulate exocytosis via P-glycoprotein (P-gp) [62], which is expressed on the plasma membrane of endothelial cells of the blood-brain barrier. According to some researchers [63, 64], high expression of *ABCB1* correlates with poor survival in GB patients. Others point to a correlation of longer survival in patients with high *ABCB1* gene expression, though its independent prognostic value was not definitively established [65]. In pancreatic and kidney tumors, high expression of *ABCB1* is a favorable prognostic factor. Increased expression of *Abcb1* was found in M2 GB and M6 GB cells. The increased expression of P-gp is likely to be caused by prolonged carcinogen exposure, facilitating active efflux of DMBA from the cells [66]. P-gp expression is regulated by several signal pathways, including PI3K/Akt. According to this study, *Pi3k* is overexpressed in M2 GB. Alterations in the PI3K pathway were detected in 17% of patients with GB [67]. Impaired regulation of PI3K transforms tumors into more aggressive and recurrent diseases, and which is consistent with our morphological and clinical observations: exhibits more malignant histology and faster grow in M2 GB compared to M6 GB, which has a wild-type *Pi3k* expression.

Expression of the *Tp53* gene, which encodes the p53 tumor suppressor and transcription factor, was elevated in the M2 GB model compared to intact brain tissue. In contrast, *Tp53* expression was decreased in the M6 GB model. In M6 GB, the combination of low *Tp53* expression and high *Pi3k* oncogene expression may drive the marked upregulation of *Hif1a*, *Vegfa*, *Gdnf*, and *Egfr*. Loss of p53 function contributes to oncogenesis in approximately 25–37% of GB. While inactivating *TP53* mutations are common in the giant cell subtype of human GB, the M2 GB model — which also exhibits giant cell morphology — shows *Tp53* overexpression, suggesting a distinct mechanism of p53 pathway dysregulation. Inactivating *TP53* mutations are also prevalent in other CNS tumors, being found in over 50% of *IDH1/2*-mutant astrocytomas and large cell medulloblastomas.

For comparison, H3K27M-mutant diffuse midline gliomas are characterized by a high frequency of *TP53* and *IDH1/2* mutations, as well as *PDGFRA* amplification. These tumors typically retain wild-type *ATRX*, while *TERT* promoter mutations and *EGFR* amplification are rare [51].

Mutations in the *TP53* gene, which lead to structural and functional alterations, are implicated in various tumor types [68]. For instance, missense *TP53* mutations are characteristic of gliomas with *IDH1/2* aberrations.

In our models, the *Cdkn2a* gene — a critical tumor suppressor, cell cycle regulator, and p53 pathway activator — was overexpressed in both M2 GB and M6 GB. This finding contrasts with the situation in human GB and WHO CNS Grade 4 astrocytoma, where homozygous deletion of *CDKN2A* is a hallmark alteration associated with high proliferative activity and poor prognosis [69].

The behavior of the p53 protein itself is complex. Exposure to carcinogens can initially increase p53 levels in non-tumor cells [70]. However, prion-like aggregation of mutant p53 can lead to its functional inactivation; these aggregates, including oligomers and amyloid- like fibrils, can sequester wild-type p53, promoting oncogenesis [71, 72]. p53 levels also critically influence cell fate: low levels facilitate stem cell formation and proliferation, while high levels promote differentiation [73]. Despite the high *Tp53* expression in M2 GB, the *Sox2* gene (a stemness marker) was also highly expressed in both models. This suggests that M6 GB tumors may be more differentiated than M2 GB, potentially due to differences in p53 activity beyond mere expression levels. Furthermore, p53 can interact with mitochondrial peptidylprolyl isomerase F (PPIF) to induce necrotic cell death [74]. This mechanism is consistent with our morphological observations, where necrosis was more pronounced and extensive in M2 GB than in M6 GB.

M2 GB and M6 GB tissues exhibited increased expression of the *Sox2* gene, a key transcription factor for stem cell maintenance and a marker of undifferentiated cells. *SOX2* overexpression is a hallmark of various poorly differentiated tumors, including GB [75]. The role of *SOX2* in tumor progression appears contextdependent. It has been demonstrated to promote cell migration and confer resistance to chemotherapy [76]. Conversely, some studies indicate that its suppression can also be associated with tumor invasion and treatment resistance, highlighting the complexity of its function. *SOX2* is highly expressed in the developing central nervous system and is essential for neural stem cell function [77]. Gliomas, similar to neural stem cells, exhibit an accessible chromatin state at the *SOX2* enhancer cluster, which contributes to its sustained high expression and drives tumor cell proliferation [78].

In contrast to the frequent PTEN deficiency observed in human GB, both M2 GB and M6 GB mouse models exhibited *Pten* overexpression. The functional consequences of *PTEN* overexpression appear to be context-dependent. While some studies demonstrate that it can induce apoptosis, disrupt mitochondrial function, and sensitize glioma cells to chemotherapy, others report that it may promote tumor cell motility and dedifferentiation in certain contexts [79–85]. The latter is supported by our finding of elevated *Cd44* and *Sox2* expression in the M6 GB model, markers associated with a less differentiated, aggressive state.

This paradox aligns with a broader concept in oncology: while tumor suppressor overexpression is typically intended to halt growth and promote differentiation or apoptosis [66], it can, under specific conditions, paradoxically drive tumor progression. Although carcinogenesis is primarily driven by the loss of tumor suppressors and activation of oncogenes, sustained overexpression of certain tumor suppressors has been linked to the progression of colorectal, breast, ovarian, head and neck cancers, and lymphomas, correlating with reduced patient survival [82, 83]. In these specific contexts, such genes may exhibit oncogenic properties.

PTEN is a known negative regulator of the PI3K/AKT/ mTOR signaling cascade, a key pathway promoting tumor cell growth. Paradoxically, both M2 GB and M6 GB models exhibited concurrent *Pten* overexpression and *Pi3k* activation. This contrasts with the situation in melanoma, where *PTEN* loss is common and associated with reduced T-cell infiltration and therapy resistance, making *PI3K* pathway inhibition a rational therapeutic strategy [84]. In our study, however, *Pten* overexpression in the M2 GB and M6 GB models was associated with substantial tumor infiltration by T-cells.

Expression of the *Mki67* gene, encoding the Ki-67 proliferation marker, was elevated in both M2 GB and M6 GB cells, consistent with the observed high mitotic activity. From a therapeutic perspective, promoter methylation of the *MGMT* gene, which silences this DNA repair enzyme, is a favorable prognostic marker in GB patients treated with alkylating agents [57]. In M2 GB and M6 GB models, *Mgmt* expression remained unchanged, suggesting intrinsic resistance to such chemotherapy. This makes these models particularly valuable for studying mechanisms of chemoresistance and developing novel antitumor strategies to overcome it.

The *VEGFA* and *HIF1α* genes are overexpressed in many human tumors, a pattern recapitulated in the M2 GB and M6 GB mouse models. VEGF-A is a key regulator of angiogenesis, controlling endothelial cell proliferation and vascular permeability; however, its function can be context-dependent, exhibiting both pro- and anti-angiogenic effects [85]. *HIF1α* is a well- established prognostic marker for predicting tumor response to treatment. In tumors, receptors for various growth factors are often constitutively active, sustaining downstream signaling even in the presence of low ligand concentrations. This dysregulated activation of receptor tyrosine kinase pathways is a major driver of tumor growth, which may represent an aberrant attempt by cancer cells to overcome stress signals that would otherwise trigger differentiation or cell death [66].

The orthologue of the *GDNF* gene, which encodes a dopaminergic neurotrophic factor and a ligand for the TGF-β superfamily, was overexpressed in M6 GB. *GDNF* can activate the SMAD transcription factor pathway, influencing cell cycle progression [86].

CD133 (prominin-1) is a marker of tumor-initiating cells in various solid tumors. Expression of its ortholog was significantly elevated in the M2 GB model. A high frequency of CD133^+^ cells is generally associated with resistance to chemo- and radiotherapy and poor patient survival [87]. However, the biology of CD133 is complex, as some differentiated cells can express it, and CD133negative cell populations can also initiate tumors [88].

*Pdgfra* levels were increased in M2 GB. *PDGFRA* activation leads to phosphorylation of PIK3R1, triggering downstream signaling cascades including calcium mobilization and activation of PKC, AKT1, HRAS/MAPK/ ERK, and STAT pathways, thereby promoting tumor growth and survival [89].

The S100B protein, a neurotrophic factor and one of the most abundant soluble proteins in the brain [90], was overexpressed in both the M2 GB and M6 GB models. S100B promotes astrocytosis and axonal growth. It exhibits a higher binding affinity for Zn^2+^ than for Ca^2+^. In glioma patients, a high serum level of S100B serves as a prognostic marker, and the S100B protein family is known to regulate glioma stem cells and mediate epithelial-mesenchymal transition in GB [91].

It is important to note that all the studied genes encode multiple protein isoforms. Mechanisms such as alternative splicing and the use of alternative promoters increase transcript diversity, leading to a variety of protein isoforms with potentially different functions [92, 93]. Furthermore, gene activity is modulated by epigenetic mechanisms and post-translational modifications. Critically, even elevated mRNA expression in tumor cells may not result in increased protein levels due to post-transcriptional repression [94, 95]. Therefore, future studies should aim to characterize the specific protein isoforms expressed and quantify their levels to fully understand their functional role in these models.

Therefore, the carcinogen DMBA induces oncogenesis in brain tissue through the formation of DNA adducts and dysregulation of genes governing key cellular processes, including angiogenesis, proliferation, invasion, development, transcription, apoptosis, DNA repair, cytoskeleton organization, metabolism, and intercellular signaling [96]. This leads to genomic instability and tumor progression. Sequential transplantation of DMBA-induced tumor tissue, along with associated tumor-associated macrophages and other microenvironmental factors, can further reshape the tumor’s genetic landscape and drive malignant progression.

Gene expression profiling of the M2 GB and M2 GB strains revealed aberrant activation of the tumor suppressors *Pten*, *Cdkn2a*, and *Tp53* in M2 GB. These alterations indicate that the compensatory cellular response to DMBA exposure — aimed at restoring DNA integrity, suppressing proliferation, facilitating carcinogen removal, and eliminating abnormal cells — was ultimately unsuccessful. The initial stages of DMBA-induced oncogenesis are characterized by non-proliferative changes, inflammation, and capsule formation [97], representing a host defense mechanism to isolate the carcinogen. Tumor cells, despite their impaired self-regulation, retain fundamental genomic regulatory features of healthy cells. Many of the genetic modifications observed in tumors may represent an adaptive response to carcinogenic stress and an attempt to compensate for defects in DNA replication [66].

The use of intracerebral tissue models like M2 GB and M6 GB in immunocompetent mice provides a valuable platform for generating new data on tumor biology, diagnosis, and therapy. These models, along with other established transplanted models such as the GB 101.8 rat glioma, play a major role in developing diagnostic tools [98–100], advancing therapeutic strategies [101], and elucidating the mechanisms of carcinogenesis [102, 103].

A primary limitation of this study is its small sample size.

## Conclusion

Experimental M2 GB and M6 GB tissue models recapitulate key features of human gliomagenesis based on clinical features of this disease (cachexia, metabolic disturbances, tumor-associated coagulopathy) and morphology (invasive aggressive growth, nuclear and cellular polymorphism, high mitotic activity, pronounced vascularization, and necrosis). Substantial immune cell infiltration (comprising up to 50% of the tumor tissue), coupled with altered expression of genes central to oncogenesis, establishes these models as highly suitable for investigating carcinogenesis and evaluating novel antitumor therapies.

Gene expression profiling revealed distinct transcriptional programs for each model. A common signature of upregulated genes in both M2 GB and M6 GB included tumor suppressors (*Cdkn2a*, *Pten*), the cellular transporter (*Abcb1*), the proliferation marker (*Mki67*), transcription factors (*Hif1a*, *Sox2*), as well as neurotrophic (*S100b*) and angiogenic (*Vegfa*) factors. Model-specific profiles were also identified: M6 GB showed unique upregulation of the growth factor receptor *Pdgfra*, the differentiation marker *Cd133*, and the tumor suppressor *Tp53*. In contrast, M2 GB was characterized by elevated expression of the signaling factor *Pi3k*, the glial filament protein *Gfap*, and the growth factor *Gdnf*.

Furthermore, comparative analysis showed that M6 GB had higher mRNA levels of genes involved in cell adhesion (*Cd44*), proliferation (*Pi3k*, *Hif1a*, *Gdnf*), and growth signaling (*Egfr*) compared to M2 GB. Conversely, M6 GB exhibited lower expression of genes related to differentiation, proliferation, and apoptosis — *Cdkn2a*, *Tp53*, and *Cd133* — as well as the *Pdgfra* receptor, relative to M2 GB.

In conclusion, the M2 GB and M6 GB models, with their human-like intratumoral immune landscape, clinical and morphological features, and distinct gene expression patterns, represent valuable tools for advancing research in gliomagenesis and for developing innovative diagnostic and therapeutic strategies.
